# Novel* GLA* Deletion in a Cypriot Female Presenting with Cornea Verticillata

**DOI:** 10.1155/2016/5208312

**Published:** 2016-03-30

**Authors:** Theodoros Georgiou, Gavriella Mavrikiou, Angelos Alexandrou, Elena Spanou-Aristidou, Isavella Savva, Theodoros Christodoulides, Maria Krasia, Violetta Christophidou-Anastasiadou, Carolina Sismani, Anthi Drousiotou, George A. Tanteles

**Affiliations:** ^1^Department of Biochemical Genetics, The Cyprus Institute of Neurology and Genetics, 2370 Nicosia, Cyprus; ^2^Department of Cytogenetics and Genomics, The Cyprus Institute of Neurology and Genetics, 2370 Nicosia, Cyprus; ^3^Clinical Genetics Department, The Cyprus Institute of Neurology and Genetics, 2370 Nicosia, Cyprus; ^4^Molecular Medicine Research Center, University of Cyprus, 1678 Nicosia, Cyprus; ^5^Cardio Health Center, 2042 Nicosia, Cyprus; ^6^Ophthalmology Centre, 2223 Nicosia, Cyprus

## Abstract

Fabry disease is an X-linked lysosomal storage disorder resulting from a deficiency of the hydrolytic enzyme *α*-galactosidase A (*α*-Gal-A). It is characterized by progressive lysosomal accumulation of globotriaosylceramide (Gb3) and multisystem pathology, affecting the skin, nervous and cerebrovascular systems, kidneys, and heart. Heterozygous females typically exhibit milder symptoms and a later age of onset than males. Rarely, they may be relatively asymptomatic throughout a normal life span or may have symptoms as severe as those observed in males with the classic phenotype. We report on a 17-year-old female in whom cornea verticillata was found during a routine ophthalmological examination but with no other clinical symptoms. Leucocyte *α*-galactosidase activity was within the overlap range between Fabry heterozygotes and normal controls. Sanger sequencing of the* GLA* gene failed to reveal any pathogenic variants. Multiplex Ligation-dependent Probe Amplification (MLPA) analysis revealed a deletion of exon 7. Using a long-range PCR walking approach, we managed to identify the deletion breakpoints. The deletion spans 1182 bp, with its 5′ end located within exon 6 of the* GLA* gene and its 3′ end located 612 bp downstream of exon 7. This finding represents a novel deletion identified in the first reported Cypriot female carrier of Fabry disease.

## 1. Introduction

Fabry disease (FD; MIM# 301500) is an X-linked lysosomal storage disorder resulting from a deficiency of the lysosomal enzyme *α*-galactosidase A (*α*-Gal-A). This deficit affects the metabolism of certain glycosphingolipids, mainly globotriaosylceramide (Gb3), that accumulate in lysosomes of many cell types.

The incidence of Fabry disease was originally reported to be between 1 : 40,000 and 170,000 [1–3], but more recent estimates from neonatal screening programs indicate a much higher incidence, 1 : 3,100 in Italy [4] and 1 : 1,500 in Taiwan [5]. The majority of the newborns detected in these two studies were found to carry mutations predicting the late-onset phenotype. Classically affected hemizygous males, with no or little residual *α*-galactosidase A activity, may display all the characteristic neurological (pain), cutaneous (angiokeratoma), renal (proteinuria, kidney failure), cardiovascular (cardiomyopathy, arrhythmia), cochlear-vestibular, and cerebrovascular (transient ischemic attacks, strokes) signs of the disease, while heterozygous females have symptoms ranging from very mild to severe [6].

One of the earliest and most distinctive ocular findings of FD is a bilateral, whorl-shaped keratopathy known as cornea verticillata which results from accumulation of Gb-3, deposited by limbal blood vessels at the level of the epithelial basement membrane and visualized as yellowish brown inclusions emanating radially from a single vortex. The cornea stroma and endothelium are spared [7]. Cornea verticillata and posterior lenticular cataracts are present in approximately 70% of carrier females and are useful in heterozygote detection [8].

The human* GLA* gene, localized at Xq22.1, is 12 kb in length and consists of seven exons [9]. To date, more than six hundred disease-causing mutations have been identified, the majority of which are missense (http://www.hgmd.cf.ac.uk/).

Management of the disease was primarily symptomatic until enzyme replacement therapy (ERT) became available for clinical use. Several clinical trials demonstrated the efficacy of ERT to prevent or slow major organ dysfunction but unable to treat certain manifestations such as angiokeratoma, hearing impairment, hypertension, and cornea verticillata [10]. Early diagnosis is of outmost importance in order to administer therapy and prevent the most harmful consequences of the disease [11].

## 2. Clinical Report

The proband, a 17-year-old female, was identified as having vortex keratopathy following routine eye examination and was subsequently referred for clinical genetics evaluation, since the possibility of FD was raised. She did complain of occasional paresthesias over her fingers and toes. There was no report of hearing, visual, or sweating abnormalities. Past medical history was otherwise unremarkable. Extensive clinical and laboratory investigation failed to reveal cutaneous, cardiac, renal, or neurological involvement. More specifically, the proband underwent a cardiac assessment which was unremarkable. There were no abnormal findings on clinical examination. On 12-lead ECG, there was sinus rhythm, with normal atrioventricular conduction, incomplete right bundle branch block (essentially normal finding), no repolarization abnormalities, and normal QT interval. On transthoracic echocardiography, there was normal cardiac structure and function. The proband underwent a 24 hr rhythm Holter recording during which she had normal sinus rhythm without any evidence of arrhythmia. There was no renal participation, as documented by the normal serum creatinine and estimated GFR (MDRD and EPI formula), the absence of proteinuria and hematuria, and the normal kidney size and echogenicity on a renal ultrasound scan. Blood pressure was repeatedly evaluated and was found to be within normal limits. Mental status was normal. There were no cutaneous stigmata. Extraocular movements were full. Pupils were equal and reactive. Visual fields were full to confrontation. There was no objective hearing loss. Sensation over the face and limbs was not impaired. Reflexes were 2+ and symmetric. There was no focal weakness or atrophy. Plantar responses were flexor bilaterally. There was no cerebellar dysfunction. Her gait was normal and Romberg test was negative. Routine biochemical and hematological test results were normal. Lyso-Gb3 in plasma was 5.9 ng/mL (normal range: 0.1–0.9 ng/mL).

## 3. Methods and Results

The activity of *α*-galactosidase in the patient's leucocytes was assayed using a standard fluorometric method [12] and was found to be 53 nmol/hr/mg protein (normal range: 33–134 nmol/hr/mg protein; carrier range: 4.3–73 nmol/hr/mg protein). The enzyme was measured in a second sample and it was again found to fall within the overlap range between FD heterozygotes and normal controls. The activity of the *β*-galactosidase enzyme was also measured as a control lysosomal enzyme and was within normal range.

**Figure 1:**
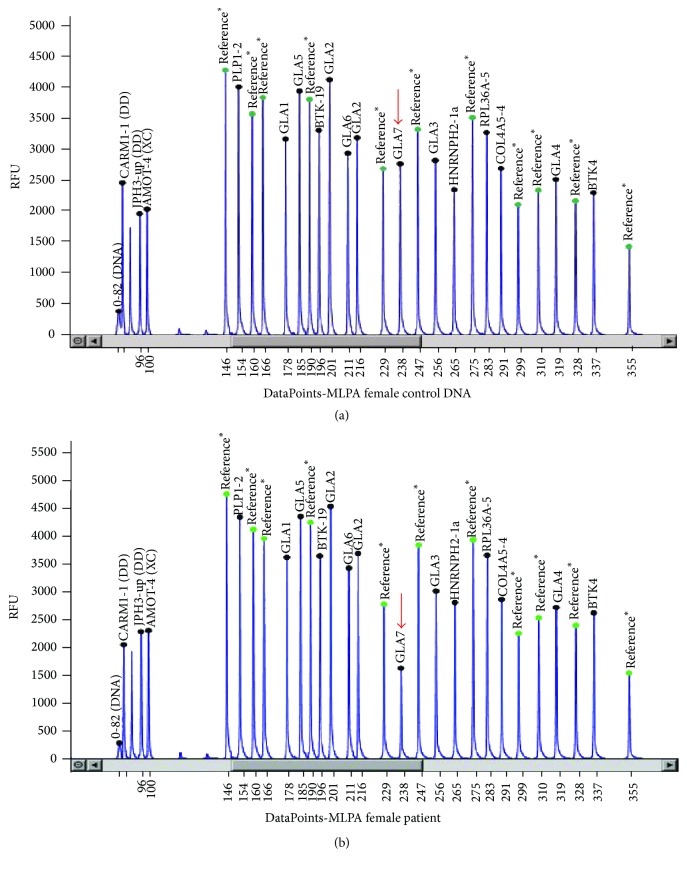
Multiplex Ligation-dependent Probe Amplification (MLPA) electropherogram tracings. (a) Normal female control and (b) proband. The arrow indicates the deleted exon 7. ^*∗*^Reference probes.

Genomic DNA was extracted from peripheral blood leucocytes using the Puregene Blood Kit (cat. number 158467). All the* GLA* gene exons, including their flanking regions, were amplified by PCR and sequenced. The primers used were those used previously as published by Morrone et al. [13]. Since no pathogenic mutations were identified by direct sequencing, Multiple Ligation-dependent Probe Amplification (MLPA) was carried out in an attempt to identify deletions or duplications using the P159 GLA probemix (MRC-Holland Amsterdam, Netherlands). MLPA was performed according to the manufacturer's protocol using 200 ng of genomic DNA per reaction. All runs included DNA from four unrelated normal controls. The patient was found to harbour an exon 7 deletion, as indicated by the average ratio of 0.51 of the corresponding probes (Figure 1). A maternal sample was also tested and found to be negative for the deletion. The father was not available for testing and we do not know whether he has any symptoms of Fabry disease.

Quantitative Real Time PCR (qRT-PCR) was carried out in the patient and the mother, in order to confirm the presence/absence of the deletion. QRT-PCR primer sequences were designed using Primer 3 software (http://bioinfo.ut.ee/primer3-0.4.0/). The primer sets were designed with an amplicon length of approximately 100 bp targeting the deleted region along with a control primer set (Metabion, Germany). For the qRT-PCR analysis, the following oligonucleotide primers were used: AA1-F 5′-GTCTCAAAGTCCCGACCTCA-3′ and AA1-R 5′-CCTGTCCACCTTTTTCTCCA-3′ (chrX: 100652650–100652767); AA2-F 5′-TTTACCCAGGGAAGCAACTG-3′ and AA2-R 5′-AGCCTGGGCTGTAGCTATGA-3′ (chrX: 100652965–100653046); Control-F 5′-GGCCCAGGACTTATCTCGAC-3′ and Control-R 5′-GTACAGGATGCCACCCCTCT-3′ (chr18: 31301854–31301978) mapped using build GRCH37 (HG19). PCR, detection, and fluorescent data analysis were carried out on the CFX96 Real-Time C1000 Thermal Cycler (Biorad Inc.) using the Sso Fast Evagreen Supermix (Biorad Inc.) according to the manufacturer's recommendations. QRT-PCR analyses using primers AA1 and AA2 confirmed the deletion in the patient and showed a normal copy number state in the mother, concordant with the MLPA results.

**Figure 2:**
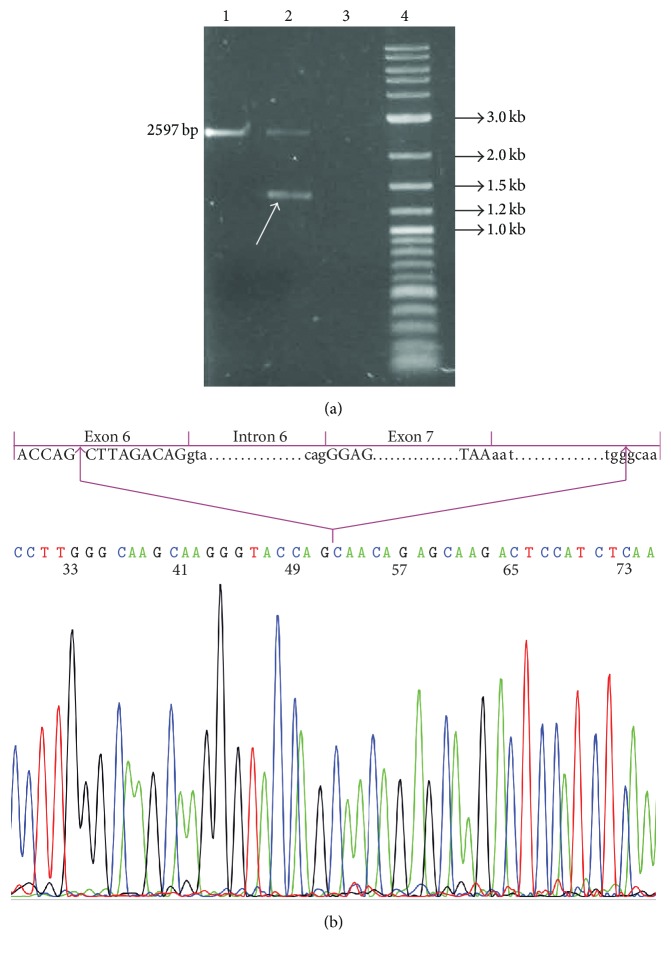
(a) Results of the long-range PCR assay in the proband with exon 7 deletion (lane 2) and healthy control (lane 1). Arrow indicates the aberrant (shorter) product. Lane 4, 10 kb ladder. (b) Breakpoint analysis and schematic figure of* GLA* gene exon 7 deletion. Sequence analysis showed a 1182 bp deletion with its 5′ end located within exon 6 and its 3′ end located 612 bp downstream of exon 7.

A long-range PCR walking strategy was used to map the deletion breakpoints. Using the pair of primers 5′-TCAAGCCAAAGCTCTCCTTC-3′ and 5′-CGCTTGTGGGGAAATTTATG-3′, regions surrounding the deleted exon 7 of the* GLA* gene were amplified by KAPALongRange DNA polymerase (KapaBiosystems, Boston, USA) according to the manufacturer's recommendations. To determine the position of the deletion breakpoints, the aberrant (shorter) long-range PCR product from the deleted allele was separated on 1% agarose gel and purified from the gel using the NucleoSpin Gel and PCR clean up kit (MACHEREY-NAGEL). The purified fragment was sequenced using the same pair of primers. The deletion was found to span 1182 bp, with its 5′ end located within exon 6 of the* GLA* gene and its 3′ end located 612 bp downstream of exon 7 (Figure 2).

The X-chromosome inactivation (XCI) pattern was determined by methylation analysis for the region flanking the polymorphic CAG repeat in exon 1 of the* AR* gene, following a modification of the method by Allen et al. [14]. X-inactivation analysis of the patient showed a random (not skewed) XCI pattern (ratio 58 : 42).

## 4. Discussion

We report the first confirmed case of Fabry disease carrier in the Cypriot population. No Cypriot male patient has been diagnosed in our laboratory during the last twenty-five years (our laboratory is the only reference laboratory for inherited metabolic disorders on the island). Furthermore, in a previous study, we screened 140 Cypriot males who had either end-stage renal disease or cardiomyopathy, of unknown etiology, by measuring *α*-galactosidase activity in peripheral blood leucocytes, and no Fabry disease patients were detected (unpublished data).

The *α*-galactosidase activity in the case presented here was within the overlap range between Fabry heterozygotes and normal controls. Deficient *α*-galactosidase activity in leukocytes or fibroblasts is commonly used to diagnose male patients, but it is a less reliable diagnostic method for female heterozygotes, as their *α*-galactosidase activity can range from normal to deficient due to nonrandom X-inactivation [15]. Hence, it is crucial to combine biochemical assays with molecular analyses to identify heterozygous FD females. However, mutation analysis using exon screening may fail to detect the mutant allele in the case of large intragenic deletions which prevent primer annealing in the deleted area. Detection of larger gene rearrangements usually requires alternative techniques such as Multiplex Ligation-dependent Probe Amplification (MLPA). This allows simultaneous hybridization and ligation of several probes in a single reaction tube, followed by PCR and analysis by capillary electrophoresis. The usefulness of MLPA in the analysis of deletions and duplications involving the* GLA* gene has been reported by other groups [16, 17]. Using MLPA, we identified a novel deletion in exon 7 in our patient which was confirmed by qRT-PCR. Our findings provide a strong rationale for screening all possible FD patients, in whom no pathogenic mutations are identified, for exonic* GLA* deletions. This is particularly important in the case of females, since it may be the only way of confirming the diagnosis. Mutation identification allows for screening for more affected individuals in the extended family and facilitates prenatal diagnosis.

We characterized the novel deletion found in our proband using long-range PCR and sequence analysis as spanning 1182 bp [g.25012_26194del1182, according to the numbering of the human* GLA *NCBI reference U78027.1], with its 5′ end located within exon 6 of the* GLA* gene and its 3′ end located 612 bp downstream of exon 7. Specifically, the deletion includes nine nucleotides before the 3′ end of exon 6, intron 6, exon 7, and 612 bp downstream of exon 7. This deletion has not been reported previously and is expected to be pathogenic, since it results in loss of part of the* GLA* coding sequence.

More than 600 pathogenic mutations (Human Gene Mutation Database, HGMD http://www.hgmd.cf.ac.uk/) have been identified so far in the human* GLA* gene. Large genomic deletions of one or more exons involving the* GLA* gene are not commonly described. However, the frequency of large deletions may be underestimated, as they escape detection by sequencing analysis. A large deletion comprising exons 5, 6, and 7 was reported in a Spanish male patient [18].

Fabry disease carriers can be affected to various degrees depending on the pattern of X-inactivation. If the X chromosome carrying the mutation is preferentially inactivated, the female will be minimally affected, whereas if the normal X chromosome is preferentially inactivated, the female may be severely affected. This correlation between the clinical phenotype and X-inactivation was found by some studies [13, 15, 19], whereas other studies found no correlation [20, 21]. X-inactivation in our proband showed a random pattern; however, it is not possible to predict what the clinical phenotype will be. On evaluation, one year after initial diagnosis, she remained asymptomatic at 18 years old. She will be followed up closely for the appearance of any symptoms and treated accordingly.

In conclusion, we would like to stress the importance of recognizing vortex keratopathy as an indication for Fabry disease, especially in female heterozygotes where other symptoms may be lacking. We would also like to emphasize the importance of applying the MLPA technique in those cases, where no mutation is identified using Sanger sequencing.
